# Novel *SACS* Mutations Identified by Whole Exome Sequencing in a Norwegian Family with Autosomal Recessive Spastic Ataxia of Charlevoix-Saguenay

**DOI:** 10.1371/journal.pone.0066145

**Published:** 2013-06-13

**Authors:** Charalampos Tzoulis, Stefan Johansson, Bjørn Ivar Haukanes, Helge Boman, Per Morten Knappskog, Laurence A. Bindoff

**Affiliations:** 1 Department of Neurology, Haukeland University Hospital, Bergen, Norway; 2 Department of Clinical Medicine, University of Bergen, Bergen, Norway; 3 Centre for Medical Genetics and Molecular Medicine, Haukeland University Hospital, Bergen, Norway; 4 Department of Biomedicine, University of Bergen, Bergen, Norway; 5 K.G. Jebsen Centre for Research on Neuropsychiatric Disorders, University of Bergen, Bergen, Norway; Oslo University Hospital, Norway

## Abstract

We employed whole exome sequencing to investigate three Norwegian siblings with an autosomal recessive spastic ataxia and epilepsy. All patients were compound heterozygous (c.13352T>C, p.Leu4451Pro; c.6890T>G, p.Leu2297Trp) for mutations in the *SACS* gene establishing the diagnosis of autosomal recessive spastic ataxia of Charlevoix-Saguenay (ARSACS). The clinical features shown by our patients were typical of this disorder with the exception of epilepsy, which is a rare manifestation. This is the first report of ARSACS in Scandinavian patients and our findings expand the genetic and clinical spectrum of this rare disorder. Moreover, we show that exome sequencing is a powerful and cost-effective tool for the diagnosis of genetically heterogeneous disorders such as the hereditary ataxias.

## Introduction

Autosomal recessive spastic ataxia of Charlevoix-Saguenay (ARSACS) is caused by mutations of the *SACS* gene, encoding sacsin, a protein that is highly expressed in neurons throughout the central nervous system [1] and apparently involved in mitochondrial fission [2]. The disease was first identified in individuals from the Quebec province in Canada where most cases are caused by two founder mutations (c.6594delT and c.5254C>T) [3], although other mutations have also been identified in this population [4]. Subsequently, cases have been described in individuals of French [5], Belgian [6], Tunisian [7], [8], Italian [9], [10], Spanish [11], Turkish [12], Dutch [13] and Japanese [14], [15] descent.

Disease onset is commonly in early childhood, but may also be later in life especially in patients originating outside Quebec. Clinical features include progressive spinocerebellar ataxia, dysarthria, nystagmus, upper motor neuron dysfunction and a distal sensorimotor peripheral neuropathy predominantly affecting the lower limbs. In addition, patients may have retinal hypermyelinated fibres appearing as yellowish retinal streaks on fundoscopy, although these are less common in individuals originating outside the province of Quebec [7], [16]. A straight dorsal spine with loss of the dorsal kyphosis was recently described in five patients [17]. MRI reveals atrophy of the cerebellum, which is most pronounced in the vermis superior, and spinal cord and linear T2 hypointensities are commonly found in the basis pontis. Cerebral atrophy may also occur later in life [18], [19].

Phenotype-genotype correlation in the hereditary spastic ataxias and paraplegias is poor. We decided, therefore, to use whole exome sequencing to establish the diagnosis in a family with a recessive spastic ataxia. We found that all three affected siblings had the same two novel heterozygous mutations in the *SACS* gene thus establishing them as the first cases of ARSACS in Scandinavia.

## Patients and Methods

The three affected siblings (one male and two females) belonged to a family that came from a small coastal community of western Norway. Neither their other 4 siblings nor their parents were reported as having any neurological disease. Printed local histories and church records covering nine generations from the parental generation were used to look for consanguinity in the family. Genome wide SNP genotyping was performed with the Genome Wide Human SNP array 6.0 (Affymetrix, Santa Clara, USA) and analysed using PLINK v1.07 [20]. For homozygozity mapping, we searched for any region >3 Mb, with minimum of 30 SNPs and less than four heterozygous calls. Whole exome sequencing was performed at HudsonAlpha Institute for Biotechnology (Huntsville,AL) using Roche-NimbleGen Sequence Capture EZ Exome v2 kit and paired-end 100nt sequencing on the Illumina HiSeq [21]. The 8.9 Giga-bases of aligned sequence data resulted in 100X median coverage of the target capture regions with more than 97% of target bases covered at least 8X. Our study was approved by the Regional Committee for Medical and Health Research Ethics, Western Norway (IRB00001872). All study participants provided written informed consent.

## Results

### Clinical Features

All three patients were born after normal pregnancies and uncomplicated deliveries. The index case, now a sixty-five year old man, had delayed motor milestones and first walked at the age of two years. Subsequently he developed progressive lower limb stiffness, gait unsteadiness, dysarthria, dysphagia, urge urinary incontinence and cognitive decline. He became wheel-chair bound in his teens. He developed complex partial motor seizures (CPM) consisting of jerking of the right upper limb; electroencephalography (EEG) data were not available. He was treated with phenytoin and later phenobarbital and remained seizure-free from his mid-twenties.

Clinical examination at the age of 54 years revealed severe dysarthria and horizontal gaze nystagmus. He had cerebellar ataxia, spastic paraparesis and a peripheral sensorimotor neuropathy with distal loss of superficial and deep sensory modalities and amyotrophy in the lower limbs. He had normal optic fundi. Electromyography (EMG) and nerve conduction velocity (NCV) studies were consistent with a predominantly axonal sensorimotor peripheral neuropathy.

**Figure 1:**
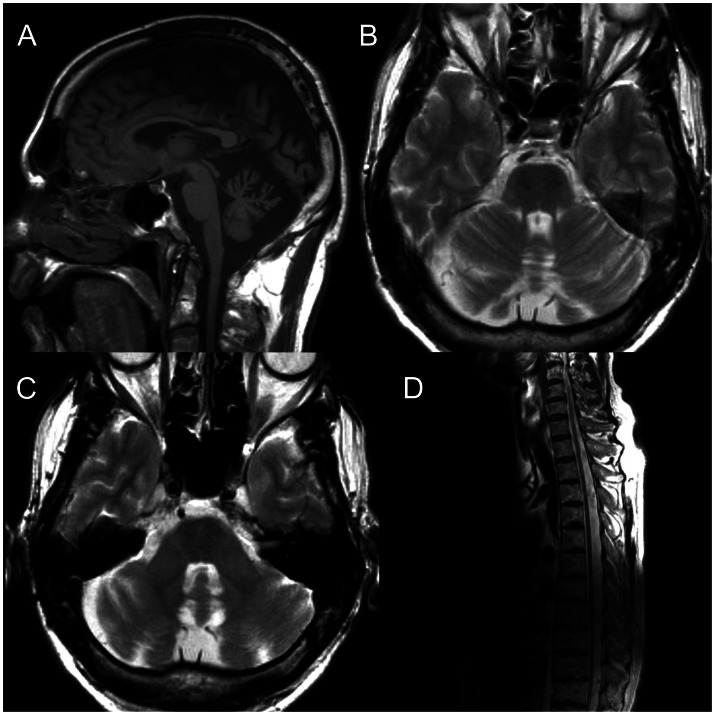
MRI of the index patient at the age of 54 years. A. Sagital T1 weighted MRI of the brain showing atrophy of the cerebellar midline, particularly the vermis superior. B&C. Axial T2 weighted MRI showing linear T2 hypointensities in the pons. C. Axial T2 weighted MRI showing prolongation of T2 signal in the dentate nuclei. D. Sagital T2 weighted MRI showing atrophy of the cord, straight dorsal spine and loss of the dorsal kyphosis.

MRI showed cerebral and cerebellar atrophy, particularly in the vermis superior and moderate atrophy of the spinal cord. There were linear T2 hypointensities in the basis pontis and T2 signal prolongation in the dentate nuclei. MRI of the spine showed a straight dorsal spine with loss of the dorsal kyphosis (Figure 1).

The index patient’s older sister, who is currently sixty-eight years old, experienced progressive gait difficulties in early childhood and went on to develop a progressive spastic ataxia with identical clinical and electrophysiological features as the index case. She developed epilepsy from the age of six years also with CPM seizures consisting of jerking of the right limbs and facial muscles. She was treated with phenytoin and phenobarbital and she remains seizure free since the age of 51 years. EEG at the age of 57 showed slow-wave (delta) activity bilaterally in the frontotemporal area, but with a significant left dominance.

The oldest affected sister, now a seventy-four year old woman, had delayed motor milestones, progressive gait difficulties and epilepsy since early childhood. Her seizures have not been evaluated by the authors, but are described as CPM with left conjugate gaze deviation and turning of the head and neck. EEG showed slow wave activity localised in the left parietotemporal area. Her clinical features and EMG findings are identical to those of her siblings.

### Genetic Findings

Information obtained from church records revealed several possible consanguineous connections between the proband’s parents. Thus, we first searched for regions of shared homozygosity among the patients. Surprisingly, genome wide SNP analysis revealed that the three affected siblings shared no regions of significant homozygosity (>2 MB). Without any candidate regions we therefore decided to sequence the exome.

**Figure 2:**
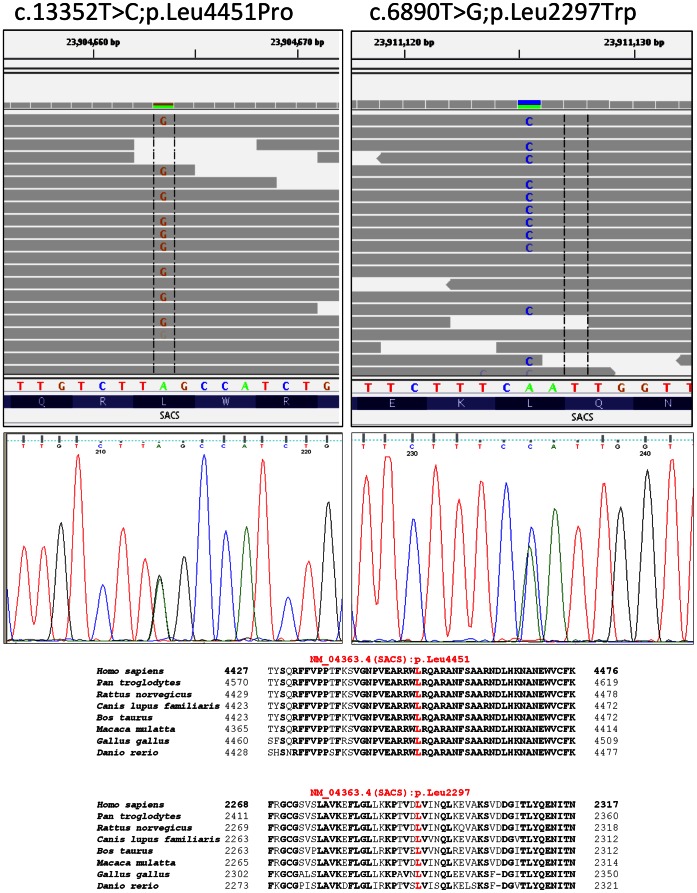
Compound heterozygous mutations found in the proband. Upper part, IGV-browser screenshots of the mutations found by exome sequencing and corresponding Sanger sequencing results. Lower part. Protein multiple sequence alignments (PMSA) of the corresponding residues generated by MUSCLE v3.6 (NCBI HomoloGene) including genes conserved in bony vertebrates (Euteleostomi). Residues in red are predicted to be affected by the mutations found in the proband.

Whole exome sequencing to a median of 100X coverage in the index patient identified 19909 genetic variants of which 9528 were non-synonymous and 206 were not found in 80 Norwegian exomes or in the 1000 Genomes database at >0.5% allele frequency. Further analysis showed that only seven genes contained rare variants consistent with autosomal recessive inheritance and of these, only one gene (*SACS*) was in a region inherited identically by all three affected siblings (Table 1). Two heterozygous mutations were found in the large exon 10 of the *SACS* gene (NCBI reference sequence NM_014363.4) and these were confirmed by Sanger sequencing (Figure 2). The c.13352T>C is predicted to cause an amino-acid substitution (p.Leu4451Pro) affecting a conserved residue located in the HEPN (Higher Eukaryotes and Prokaryotes Nucleotide-binding) domain (Figure 2). The c.6890T>G is predicted to cause a substitution at another conserved site, p.Leu2297Trp. Both mutations are predicted to be deleterious by PolyPhen-2, SIFT and MutationTaster, and are not found in the 1000Genomes database or dbSNP, nor in 186 local blood donors. The p.Leu2297Trp change was found in one of the healthy sisters confirming that the two mutations were in trans in our patients.

**Table 1:** Variant filtration of exome sequencing data from the index case compared with whole genome genotyping data in all three affected siblings.

Filter	Count
Exomic variants	19909
Excluding synonymous	9528
Not in 80 Norwegian exomes	243
Not in 1000Genomes (0.5% MAF)	206*
Putative autosomal recessive genes	7
Shared by all three siblings	1

Only one gene, *SACS*, harbors variants consistent with autosomal recessive inheritance and shared by all three siblings.

* 166 variants were not listed in dbSNP.

## Discussion

We used exome sequencing to identify the disease causing mutations in this family with an autosomal recessive spinocerebellar ataxia with upper motor neuron dysfunction. This clinical presentation has a broad differential diagnosis including several ataxia and HSP syndromes and with a long list of putative genes, testing them individually by Sanger sequencing would be a lengthy and costly process. By using exome sequencing, we identified the mutations using only a fraction of the time and cost of a conventional analysis approach.

Filtering of the exome data against control databases and by phenotype segregation left us with a single gene candidate (*SACS*) in which we found two previously unreported mutations. The patients’ phenotype is consistent with ARSACS and the mutations are predicted to have a damaging effect on the protein (sacsin). We therefore concluded that the mutations are disease-causing in this family.

This is the second report of ARSACS diagnosed by exome sequencing. Our findings, and those of other recently published studies [22]–[24], show that exome sequencing is an effective diagnostic tool for hereditary ataxias and other Mendelian disorders. The technique does have limitations, however, including not detecting non-coding variants located distant to the exons nor will it detect large deletions and duplications, and repeat expansions. The majority of Mendelian disease appears, however, to be caused by coding point mutations or splice mutations affecting the intronic-exonic boundaries. Therefore, exome sequencing is a powerful and cost-effective tool for the diagnosis of Mendelian disorders with heterogeneous genetic aetiology.

This is the first report of ARSACS in a Scandinavian family [25]. Our patients had typical disease traits including early onset, slowly progressive spastic spinocerebellar ataxia and sensorimotor peripheral neuropathy. In addition to these well-known manifestations, all three of our patients had epilepsy with CPM seizures. While EEG abnormalities are common in patients with ARSACS [26], epilepsy is a rare feature seen in about 7% of the patients [18].

No evidence of retinal hypermyelinated fibres was found in our patients, which is in line with the observation that these are uncommon in individuals originating outside the province of Quebec. MRI findings in the index case were consistent with ARSACS including the straight dorsal spine and loss of the dorsal kyphosis which was recently described in five patients [17]. In addition there was T2 signal prolongation in the dentate nucleus, which has not been previously described in this disorder. The combination of cerebellar cortical atrophy and signal change in the dentate is consistent with cerebellofugal degeneration (i.e. loss of cerebellar efferent connections) and is seen in other disorders with ataxia such as cerebrotendinous xanthomatosis [27] and the syndrome of mitochondrial spinocerebellar ataxia and epilepsy (MSCAE) [28]. While severe Purkinje cell loss has been found in post-mortem examination of ARSACS patients, it is noteworthy that no pathology has been described in the dentate nucleus. Neuropathological analysis has only been reported in two cases however, both of which originated from the province of Quebec [29], [30]. Our findings raise the possibility that dysfunction of the dentate nucleus may also occur and contribute to the pathogenesis of ataxia in patients with ARSACS.

## References

[1] Engert JC, Berube P, Mercier J, Dore C, Lepage P, et al (2000) ARSACS, a spastic ataxia common in northeastern Quebec, is caused by mutations in a new gene encoding an 11.5-kb ORF. Nature Genetics 24: 120–125. 10655055 10.1038/72769

[2] Girard M, Lariviere R, Parfitt DA, Deane EC, Gaudet R, et al (2012) Mitochondrial dysfunction and Purkinje cell loss in autosomal recessive spastic ataxia of Charlevoix-Saguenay (ARSACS). Proc Natl Acad Sci U S A 109: 1661–1666. 22307627 10.1073/pnas.1113166109 PMC3277168

[3] Richter A, Rioux JD, Bouchard JP, Mercier J, Mathieu J, et al (1999) Location score and haplotype analyses of the locus for autosomal recessive spastic ataxia of Charlevoix-Saguenay, in chromosome region 13q11. Am J Hum Genet 64: 768–775. 10053011 10.1086/302274 PMC1377794

[4] Thiffault I, Dicaire MJ, Tetreault M, Huang KN, Demers-Lamarche J, et al (2013) Diversity of ARSACS mutations in French-Canadians. Can J Neurol Sci 40: 61–66. 23250129 10.1017/s0317167100012968

[5] Anheim M, Chaigne D, Fleury M, Santorelli FM, De Seze J, et al (2008) [Autosomal recessive spastic ataxia of Charlevoix-Saguenay: study of a family and review of the literature]. Rev Neurol (Paris) 164: 363–368. 18439928 10.1016/j.neurol.2008.02.001

[6] Ouyang Y, Segers K, Bouquiaux O, Wang FC, Janin N, et al (2008) Novel SACS mutation in a Belgian family with sacsin-related ataxia. J Neurol Sci 264: 73–76. 17716690 10.1016/j.jns.2007.07.022

[7] Mrissa N, Belal S, Hamida CB, Amouri R, Turki I, et al (2000) Linkage to chromosome 13q11–12 of an autosomal recessive cerebellar ataxia in a Tunisian family. Neurology 54: 1408–1414. 10751248 10.1212/wnl.54.7.1408

[8] El Euch-Fayache G, Lalani I, Amouri R, Turki I, Ouahchi K, et al (2003) Phenotypic features and genetic findings in sacsin-related autosomal recessive ataxia in Tunisia. Arch Neurol 60: 982–988. 12873855 10.1001/archneur.60.7.982

[9] Criscuolo C, Banfi S, Orio M, Gasparini P, Monticelli A, et al (2004) A novel mutation in SACS gene in a family from southern Italy. Neurology 62: 100–102. 14718706 10.1212/wnl.62.1.100

[10] Grieco GS, Malandrini A, Comanducci G, Leuzzi V, Valoppi M, et al (2004) Novel SACS mutations in autosomal recessive spastic ataxia of Charlevoix-Saguenay type. Neurology 62: 103–106. 14718707 10.1212/01.wnl.0000104491.66816.77

[11] Criscuolo C, Sacca F, De Michele G, Mancini P, Combarros O, et al (2005) Novel mutation of SACS gene in a Spanish family with autosomal recessive spastic ataxia. Mov Disord 20: 1358–1361. 16007637 10.1002/mds.20579

[12] Richter AM, Ozgul RK, Poisson VC, Topaloglu H (2004) Private SACS mutations in autosomal recessive spastic ataxia of Charlevoix-Saguenay (ARSACS) families from Turkey. Neurogenetics 5: 165–170. 15156359 10.1007/s10048-004-0179-y

[13] Vermeer S, Meijer RP, Pijl BJ, Timmermans J, Cruysberg JR, et al (2008) ARSACS in the Dutch population: a frequent cause of early-onset cerebellar ataxia. Neurogenetics 9: 207–214. 18465152 10.1007/s10048-008-0131-7 PMC2441586

[14] Ogawa T, Takiyama Y, Sakoe K, Mori K, Namekawa M, et al (2004) Identification of a SACS gene missense mutation in ARSACS. Neurology 62: 107–109. 14718708 10.1212/01.wnl.0000099371.14478.73

[15] Shimazaki H, Sakoe K, Niijima K, Nakano I, Takiyama Y (2007) An unusual case of a spasticity-lacking phenotype with a novel SACS mutation. J Neurol Sci 255: 87–89. 17349660 10.1016/j.jns.2007.02.002

[16] Bouchard JP, Barbeau A, Bouchard R, Bouchard RW (1978) Autosomal recessive spastic ataxia of Charlevoix-Saguenay. Can J Neurol Sci 5: 61–69. 647499

[17] Gazulla J, Benavente I, Vela AC, Marin MA, Pablo LE, et al (2012) New findings in the ataxia of Charlevoix-Saguenay. J Neurol 259: 869–878. 21993619 10.1007/s00415-011-6269-5

[18] Bouchard JP, Richter A, Mathieu J, Brunet D, Hudson TJ, et al (1998) Autosomal recessive spastic ataxia of Charlevoix-Saguenay. Neuromuscul Disord 8: 474–479. 9829277 10.1016/s0960-8966(98)00055-8

[19] Martin MH, Bouchard JP, Sylvain M, St-Onge O, Truchon S (2007) Autosomal recessive spastic ataxia of Charlevoix-Saguenay: a report of MR imaging in 5 patients. AJNR Am J Neuroradiol 28: 1606–1608. 17846221 10.3174/ajnr.A0603 PMC8134385

[20] Purcell S, Neale B, Todd-Brown K, Thomas L, Ferreira MA, et al (2007) PLINK: a tool set for whole-genome association and population-based linkage analyses. Am J Hum Genet 81: 559–575. 17701901 10.1086/519795 PMC1950838

[21] Haugarvoll K, Johansson S, Tzoulis C, Haukanes BI, Bredrup C, et al (2013) MRI characterisation of adult onset alpha-methylacyl-coA racemase deficiency diagnosed by exome sequencing. Orphanet J Rare Dis 8: 1. 23286897 10.1186/1750-1172-8-1 PMC3567975

[22] Sailer A, Scholz SW, Gibbs JR, Tucci A, Johnson JO, et al (2012) Exome sequencing in an SCA14 family demonstrates its utility in diagnosing heterogeneous diseases. Neurology 79: 127–131. 22675081 10.1212/WNL.0b013e31825f048e PMC3390538

[23] Ku CS, Cooper DN, Polychronakos C, Naidoo N, Wu M, et al (2012) Exome sequencing: dual role as a discovery and diagnostic tool. Ann Neurol 71: 5–14. 22275248 10.1002/ana.22647

[24] Pyle A, Horvath R, Chinnery PF (2012) Autosomal recessive spastic ataxia of charlevoix-saguenay in the time of next-generation sequencing-reply. Arch Neurol 69: 1661–1662. 23229046 10.1001/2013.jamaneurol.70

[25] Erichsen AK, Koht J, Stray-Pedersen A, Abdelnoor M, Tallaksen CM (2009) Prevalence of hereditary ataxia and spastic paraplegia in southeast Norway: a population-based study. Brain 132: 1577–1588. 19339254 10.1093/brain/awp056

[26] Bouchard RW, Bouchard JP, Bouchard R, Barbeau A (1979) Electroencephalographic findings in Friedreich's ataxia and autosomal recessive spastic ataxia of Charlevoix-Saguenay (ARSACS). Can J Neurol Sci 6: 191–194. 487309 10.1017/s0317167100119626

[27] Vanrietvelde F, Lemmerling M, Mespreuve M, Crevits L, De Reuck J, et al (2000) MRI of the brain in cerebrotendinous xanthomatosis (van Bogaert-Scherer-Epstein disease). Eur Radiol 10: 576–578. 10795535 10.1007/s003300050964

[28] Tzoulis C, Neckelmann G, Mork SJ, Engelsen BE, Viscomi C, et al (2010) Localized cerebral energy failure in DNA polymerase gamma-associated encephalopathy syndromes. Brain 133: 1428–1437. 20400524 10.1093/brain/awq067

[29] Bouchard JP (1991) Recessive Spastic Ataxia Of Charlevoix-Saguenay. Vinken, P J, G W Bruyn and H L Klawans. 451–459.

[30] Richter A, Morgan K, Bouchard JP, Mathieu J, Lamarche J, et al (1996) ARSACS: Possibly a lysosomal storage disease? American Journal of Human Genetics 59: A379.

